# Alcohol use and associated factors among women attending antenatal care in Southern Ethiopia: a facility based cross sectional study

**DOI:** 10.1186/s13104-019-4703-4

**Published:** 2019-10-24

**Authors:** Birhanie Mekuriaw, Zelalem Belayneh, Tinsae Shemelise, Robel Hussen

**Affiliations:** 1 Department of Psychiatry, College of Health and Medical Science, Dilla University, Dilla, Ethiopia; 2 Department of Public Health, College of Health and Medical Science, Dilla University, Dilla, Ethiopia

**Keywords:** Alcohol use, Pregnancy, Women, Anti natal Care, Gedeo zone, Harm full drinking

## Abstract

**Objectives:**

The aim of this study was to assess the prevalence and associated factors of alcohol use among women attending Antenatal Care in Gedeo zone rural health centers (Southern Ethiopia). This was a cross-sectional study conducted among randomly selected 718 pregnant women attending Antenatal Care. Alcohol Use Disorder Identification Test-C was used to assess alcohol consumption. Variables with p-values of < 0.05 in the multivariable logistic regression were considered as having a statistically significant association with alcohol use.

**Results:**

The prevalence of alcohol use among pregnant women attending antenatal care service was 8.1% with 95% CI (6.3–10.0). Unplanned pregnancy [AOR = 2.12, 95% CI (1.20, 3.73)], abortion history [AOR = 2.40, 95% CI (1.16, 4.96)], pre pregnancy alcohol use [AOR 2.17, 95% CI (1.18, 4.00)] and mental distress [AOR = 3.50, 95% CI (1.99, 6.15)] were variables found to have a statistically significant association with alcohol use. This calls a holistic and multi modal approach for the prevention, early identification and intervention of alcohol use during pregnancy. More emphasis should also be given for pregnant women with unplanned pregnancy, history of abortion, pre pregnancy alcohol use and mental distress.

## Introduction

A couple of decades have been counted since alcohol use is identified as a global burden of disease with mortality rate of more than chronic medical conditions like HIV and tuberculosis [1]. Alcohol consumption has been identified as a critical problem among pregnant women and child bearing mothers dating back to very ancient time. This can significantly affect the health status of both the mother and her babies in almost all socioeconomic groups [2, 3].

Studies showed that binge alcohol drinking (drinking five and above units of drink in one occasion) is harmful for the mother and the developing fetus due to the destructive or teratogenic effects as well as higher vulnerability of fetal alcohol spectrum disorders [4, 5]. Although multiple clinical recommendations and public health campaigns are invested regarding the problems of alcohol use during pregnancy, evidences showed that pregnant women continue to consume alcohol across the world, particularly in low-income countries like Ethiopia [6–8].

World health organization (WHO) estimates the prevalence of alcohol consumption during pregnancy as 7.9% and 3.4–20.5% in Ethiopia and east Africa, respectively [7]. The prevalence of alcohol use among pregnant women was reported to be 15.1% in Tanzania [9], 34% in Ethiopia [10], 59.3% in Nigeria [11] and 20.4% in Ghana [12]. Partner alcohol drinking, multi parity, unplanned pregnancy, partner violence, history of still birth, abortion history and poor social support were some of the factors contributing for alcohol use during pregnancy [9, 10, 12–14].

Literatures also revealed that women might use alcohol as a coping mechanism to escape from pregnancy related stressful situations and emotional instability [15, 16]. The drinking habits of alcohol can be also determined by the culture, attitude or other socio-demographic contexts of the society in which the pregnant mother belongs [17, 18].

In Ethiopia, there are different locally made and culturally accepted alcoholic beverages (Tella, Tej, Areki, wine….) with different alcoholic contents. These alcoholic beverages are commonly consumed in daily basis during meals. Thus, people use such easily accessible and culturally acceptable beverages in different parties, ceremonies and even, in daily family meals [19, 20].

However, little is known regarding the prevalence and associated factors of alcohol use among pregnant women attending ANC service. Therefore, it is vital to screen alcohol use and identify its associated factors among women attending ANC service at rural health centers.

## Main text

### Methods

#### Study design and period

This was an institutional based cross sectional study conducted among women attending antenatal care in Gedeo zone rural health centers from June 1st to August 1st 2017.

#### Study setup

The study was conducted at Gedeo zone rural health centers. Gedeo zone is found in Southern Nation Nationalities and Peoples Regional (SNNPR) states of Ethiopia at 359 km Sothern from Addis Ababa (the capital city of Ethiopia). The total population size of the zone is 847,434 (424,742 male and 422,692 female) according to the 2007 population and housing census [21]. There are about 41,733 pregnant women estimated to initiate ANC1 within rural health centers of the zone annually.

#### Sample size and sampling technique

The sample size was determined using a single population proportion formula. We used assumptions of prevalence of alcohol use (p = 34%) from a study done in North-west part of Ethiopia (Bahir Dar) [10], 5% margin of error and 95% confidence interval. Considering 10% non response rate and design effect of two, the total sample size was 759.

Two-stage sampling technique was used to select study subjects. First, four woredas were randomly selected from a total of six woredas and two city administrations. In the second stage, two health centers were selected from each of the selected four woredas through lottery method. Then, the calculated sample size was proportionally allocated to each of eight randomly selected health centers. Finally, pregnant women attending ANC service were interviewed consecutively until the allocated sample size was addressed.

#### Data collection procedures and instruments

A pre-tested interviewer administered questionnaire was used for the data collection. The questionnaire had different components including socio demographic characteristics, obstetric and gynecological factors, substance related variables, Oslo-3 Social Support Scale, Self Reporting Questionnaire (SRQ-20) and Alcohol Use Disorder Identification Test-C (AUDIT-C).

Alcohol use was measured using AUDIT-C [22, 23] derived from the 10-item alcohol use disorder identification test which is cross culturally validated and preferred tool to measure alcohol consumption [24, 25]. AUDIT-C has been used in different studies to measure alcohol use among pregnant women [9, 26, 27]. It has three Likert scale questions used to evaluate the frequency and quantity of alcohol intake. AUDIT-C has a total sum score of 12 which can be categorized as low risk (1–3), moderate risk (4–5) and high risk (≥ 6). The questions of AUDIT-C were adapted for pregnancy and contextualized for the study area. In this study, women with sum scores of greater and equal to three were considered alcohol user.

Level of social support was assessed using Oslo-3 item Social Support Scale having a maximum sum score of 14. The sum scores were categorized as poor (0–3), moderate (9–11) and strong (12–14) [28]. An Ethiopian validated Self Reporting Questionnaire (SRQ-20) was used to screen mental distress. It shows god accuracy to screen mental distress in primary health care settings and communities of low-income countries with a cut-off point of 7 and above [29].

The questionnaire was first prepared in English and translated to Amharic and Gedeou’fa (commonly spoken languages in the study area). Back translation to English was done to check its consistency. Pre test was done on 5% (n = 43) of the sample among women attending ANC service at Chucko heath center. The data were collected by nine degree level health professionals supervised by three masters level public health professionals after attending 3 days of training regarding the contents of the questionnaire and data collection procedures.

#### Data analysis

First, the collected data were checked for its completeness and consistency and entered to Epi-INFO (software). Then, the data were exported to a Statistical Package for Social Science (SPSS-version 20) for analysis. Descriptive analysis was used to determine the prevalence of alcohol use and its distribution among the characteristics of participants. Both bivariable and multivariable binary logistic regression were computed to identify factors associated with alcohol use. Variables with p-values of ≤ 0.25 in the bivariable analysis were considered as candidates for multivariable regression to control possible confounders. In the final model, variables with p-values of < 0.05 were considered as having a statistically significant association with alcohol use at a corresponding 95% CI.

### Results

#### Socio-demographic characteristics

Among a total of 759 women invited to participate, 718 completed the interview with a response rate of 94.6%. More than half, (53.5%; n = 384) of participants were within the age range of 25–29 years old and the mean (standard deviation) age of respondents was 27.1 (± 4.23) year. Large proportion, (77.6%; n = 557) of participants were married and living together. More than half (57.4%; n = 412) of the participants were Gedeo in ethnicity (Table 1).

**Table 1:** Socio-demographic characteristics of pregnant women attending Ante Natal Care in Gedeo Zone Health Centers, Southern Ethiopia, 2017 (n = 718)

Variables	Categories	Frequency	Percentage
Age in years	≤ 20	42	5.8
	21–24	155	21.6
	25–29	384	53.5
	≥ 30	137	19.1
Marital status	Married	557	77.6
	Single	161	22.4
Religion	Protestant	413	57.5
	Orthodox	201	28.0
	Muslim	73	10.2
	Others^a^	31	4.3
Ethnicity	Gedeo	412	57.4
	Wolaita	93	13.0
	Gurage	83	11.6
	Oromo	86	12.0
	Others^b^	44	6.1
Residency	Urban	326	45.4
	Rural	392	54.6
Occupation	Farmers	241	33.6
	Employed	166	23.1
	Merchant	121	16.9
	House wife	68	9.5
	Others^c^	122	17.0
Educational level	Unable to read and write	218	30.4
	Able to read and write	171	23.8
	Primary school	125	17.4
	Secondary school	109	15.2
	College and above	95	13.2
Monthly income in ETB	≤ 500	147	20.5
	501–999	159	22.1
	1000–1999	332	46.2
	≥ 2000	80	11.1

^a^Other religions—catholic and wakifeta

^b^Other ethnicities—Amhara and Sidama

^c^Other occupations—daily laborer and students

#### Obstetrics and psychosocial factors

Regarding the gestational age, 52.2% (n = 375) were within the third trimester. Of the total participants, 19.2% (n = 142) had pre pregnancy alcohol use history. About 26.2% (n = 188) of participants were screened positive for mental distress (Table 2).

**Table 2:** Obstetric, substance and psychosocial related factors among pregnant women attending Ante Natal Care in Gedeo Zone Health Centers, Southern Ethiopia, 2017 (n = 718)

Variables	Categories	Frequency	Percentage
Gestational age	First trimesters	121	16.9
	Second trimesters	22	30.9
	Third trimesters	375	52.2
No. of children	No child yet	298	41.5
	Has one child	127	17.7
	Has two child	133	18.5
	Has three and more	160	22.3
Abortion history	Yes	82	11.4
	No	636	88.6
History of still birth	Yes	69	9.6
	No	649	90.4
Alcohol use before pregnancy	Yes	142	19.2
	No	576	80.8
Partner alcohol use	Yes	377	46.9
	No	381	53.1
Current khat chewing	No	647	90.1
	Yes	71	9.9
Information regarding substance problem	Yes	115	16.0
	No	603	84.0
Mental distress	Negative	530	73.8
	Positive	188	26.2
Social support level	Poor	232	32.3
	Moderate	237	33
	Strong	249	34.7

#### Prevalence and associated factors of alcohol use

The overall prevalence of alcohol use was found to be 8.1% (n = 58) with 95% CI (6.3, 10.0). During bivariable regression, age, place of residency, number of children, pregnancy plan, pre pregnancy alcohol use, abortion history, khat chewing, mental distress and level of social support were variables associated with alcohol use. After adjusting for possible confounders, unplanned pregnancy, abortion history, pre pregnancy alcohol use and mental distress were found to have a statistically significant association with alcohol use (Table 3).

**Table 3:** Variables associated with alcohol use among pregnant women attending Ante Natal Care in Gedeo Zone Health Centers, Southern Ethiopia, 2017

Variables	Alcohol use status		COR (95% CI)	AOR (95% CI)	p-value
	Yes	No			
Ages in years					
≤ 20	5 (11.9%)	37 (88.1%)	1.00	1.00	1.00
21–24	9 (5.8%)	146 (94.2%)	0.45 (0.14–1.44)	0.50 (0.14–1.73)	0.280
25–29	32 (8.3%)	352 (91.3%)	0.67 (0.24–1.83)	0.74 (0.25–2.19)	0.580
≥ 30	12 (8.8%)	125 (91.2%)	0.71 (0.23–2.14)	0.78 (0.23–2.60)	0.680
Residency					
Urban	34 (10.4%)	292 (89.6%)	1.78 (1.03–3.07)	1.71(0.97–3.01)	0.062
Rural	24 (6.1%)	368 (93.9%)	1.00	1.00	1.00
Pregnancy plan					
Planned	27 (6.0%)	424 (94.0%)	1.00	1.00	1.00
Unplanned	31 (11.6%)	236 (88.4%)	2.06 (1.20–3.54)	2.12 (1.20–3.73)	0.007*
No of children					
No children yet	17 (5.7%)	281 (94.3%)	1.00	1.00	1.00
Has one child	13 (10.2%)	114 (89.8%)	1.88 (0.88–4.00)	1.74 (0.78–3.86)	0.169
Has two child	13 (9.8%)	120 (90.2%)	1.79 (0.84–3.80)	1.33 (0.60–2.96)	0.471
Three and more	15 (9.4%)	145 (90.6%)	1.71 (0.83–3.52)	1.67 (0.77–3.62)	0.190
Abortion history					
Yes	12 (14.6%)	0 (85.4%)	2.19 (1.11–4.34)	2.40 (1.16–4.96)	0.018*
No	46 (7.2%)	590 (92.8%)	1.00	1.00	1.00
Pre pregnancy alcohol use					
Yes	19 (13.4%)	123 (86.6%)	2.12 (1.18–3.80)	2.17 (1.17–4.00)	0.013*
No	39 (6.8%)	537 (93.2%)	1.00	1.00	1.00
Khat chewing					
No	46 (7.1%)	601 (92.9%)	1.00	1.00	1.00
Yes	12 (16.9%)	59 (83.1%)	2.65 (1.33–5.29)	1.83 (0.87–3.87)	0.110
Mental distress					
Negative	29 (5.5%)	501 (94.5%)	1.00	1.00	1.00
Positive	29 (15.4%)	159 (84.6%)	3.15 (1.82–5.43)	3.50 (1.99–6.15)	0.000*
Social support level					
Poor	21 (9.1%)	211 (90.9%)	1.55 (0.78–3.09)	1.49 (0.72–3.05)	0.275
Moderate	22 (9.3%)	215 (90.7%)	1.59 (0.80–3.15)	1.40 (0.67–2.90)	0.360
Strong	15 (6.0%)	234 (94.0%)	1.00	1.00	1.00

* Significantly associated variables at p-value < 0.05, 1.00—References

### Discussion

The prevalence of alcohol use was found to be 8.1% (n = 58) with 95% CI (6.3%-10.0%). The magnitude of alcohol use in this study was in line with WHO estimate of alcohol use during pregnancy in Ethiopia(7.9%) [7]. However, the prevalence of alcohol use in this study was lower than the prevalence of alcohol use reported from a study of Northwest Ethiopia (34%) [10]. The possible explanation for this difference might be due to the difference in the screening tools used. The study conducted in Bahir-Dare used “T-ACE” which is more sensitive than AUDIT-C to screen alcohol use and might overestimate the prevalence [26]. In addition, the study conducted at Bahir-Dare was a community based study that can address all pregnant women, even not attending ANC follow-up. However, our study included only pregnant women attending ANC clinic. Inclusion of only pregnant women attending ANC clinic might yield a lower prevalence of alcohol use as women attending ANC can have the opportunity of counseling service, and may gain better knowledge and attitude towards the health of the fetus and themselves. Furthermore, women with substance use including alcohol might under report their consumption while they are at ANC service due to the fear of breaking professional advice of “not taking any alcohol during pregnancy”. The prevalence of alcohol use in this study was also lower than other studies of Tanzania (15.1%) [9], Ghana (20.4%) [12] and Nigeria (59.3%) [11]. The possible reason for this discrepancy might be due to the differences of screening tools used to measure alcohol use as well as the cultural and socio-demographic variation of study participants.

This study also identified correlates of alcohol use among women attending ANC service. Accordingly, unplanned pregnancy, pre pregnancy alcohol use, abortion history and mental distress were found to have a statistically significant association. The odds of alcohol use among women with unplanned pregnancy were 2.1 times higher as compared to their counterparts. This is consistent with other studies of Bahir-Dare and Korea [10, 27]. The possible explanation for this correlation might be due the social and psychological crisis of unplanned pregnancy that can have a potential to push pregnant women for the initiation of alcohol or other substance use to get relief from their stress [30].

Similarly, the odds of having alcohol use among pregnant women who had pre pregnancy alcohol use history were 2.2 times higher than women who had not history of alcohol use before their current pregnancy. This finding is in agreement with a recent review and another similar study which identified pre pregnancy alcohol use as a precipitant for alcohol consumption during pregnancy [14, 27]. Moreover, the brain rewarding systems of alcohol has a tendency to develop dependency which might be difficult to abstain or decrease drinking, and enforce to search alcoholic beverages even during pregnancy period [31]. Pregnant women who had history of abortion had 2.4 times increased odds of being alcohol user as compared to women who had not abortion history. This might be explained by the fact that previous abortion can elevate rates of substance use and other mental health problems like depression and anxiety [32]. However, a study conducted in Tanzania showed a reverse result indicating complications in previous pregnancy as a protective factor of alcohol use during pregnancy [9]. The possible reason for this discrepancy might be due to the fact that some women may associate previous pregnancy complications with the negative effect of alcohol that might help them to cease drinking for their future pregnancy. On the contrary, others pregnant women may develop psychological distress following pregnancy related complication which can push them to use alcohol to escape from stressful situation. Furthermore, it needs further investigation in this regard.

Pregnant women with mental distress were 3.5 times more likely to use alcohol as compared to their counterparts. This might be due to the fact that people with mental distress are more like to use alcohol to reverse the difficulties of sleep initiation, social engagement and lack of happiness which are hallmark symptoms of mental distress [33, 34].

## Limitations of the study

Participants were recruited from ANC clinics which might not be representative for women who do not attend ANC. Therefore, further community based study is recommended to address the level of alcohol use among women who do not attend ANC service. The cross-sectional nature of the study design might not show the cause and effect relationships between alcohol use and other variables.

## Data Availability

All the data included in this manuscript can be accessed from the corresponding author Birhanie Mekuriaw upon request through the email address of “biradilla@gmail.com”.

## Abbreviations

ANC: antenatal care
AOR: adjusted odd ratio
CI: confidence interval
AUDIT-C: Alcohol Use Disorder Identification Test-Consumption
COR: crude odd ratio
DURH: Dilla University Referral Hospital
ETB: Ethiopian Birr
SD: standard deviation
SRQ: Self Reporting Questionnaire
SSNPR: Southern Nation Nationalities and Peoples of Region
T-ACE: (Tolerance, Annoyed, Cut off and Eye opening)
WHO: World Health Organization
